# The non-receptor tyrosine kinase TNK2/ACK1 is a novel therapeutic target in triple negative breast cancer

**DOI:** 10.18632/oncotarget.13579

**Published:** 2016-11-25

**Authors:** Xinyan Wu, Muhammad Zahari Saddiq, Santosh Renuse, Dhanashree S Kelkar, Mustafa A Barbhuiya, Pamela L Rojas, Vered Stearns, Edward Gabrielson, Pavani Malla, Saraswati Sukumar, Nupam P Mahajan, Akhilesh Pandey

**Affiliations:** ^1^ Department of Biological Chemistry, Johns Hopkins University School of Medicine Baltimore, MD 21205, U.S.A; ^2^ McKusick-Nathans Institute of Genetic Medicine, Johns Hopkins University School of Medicine Baltimore, MD 21205, U.S.A; ^3^ Department of Oncology, Johns Hopkins University School of Medicine Baltimore, MD 21205, U.S.A; ^4^ Department of Pathology, Johns Hopkins University School of Medicine Baltimore, MD 21205, U.S.A; ^5^ Institute of Bioinformatics, International Technology Park, Bangalore, 560066, India; ^6^ Department of Drug Discovery, Moffitt Cancer Center, Tampa, FL 33612, U.S.A; ^7^ Department of Oncologic Sciences, University of South Florida, Tampa, FL 33612, U.S.A

**Keywords:** TNK2, triple negative breast cancer, tyrosine kinase, phosphorylation

## Abstract

Breast cancer is the most prevalent cancer in women worldwide. About 15-20% of all breast cancers do not express estrogen receptor, progesterone receptor or HER2 receptor and hence are collectively classified as triple negative breast cancer (TNBC). These tumors are often relatively aggressive when compared to other types of breast cancer, and this issue is compounded by the lack of effective targeted therapy. In our previous phosphoproteomic profiling effort, we identified the non-receptor tyrosine kinase TNK2 as activated in a majority of aggressive TNBC cell lines. In the current study, we show that high expression of TNK2 in breast cancer cell lines correlates with high proliferation, invasion and colony forming ability. We demonstrate that knockdown of TNK2 expression can substantially suppress the invasiveness and proliferation advantage of TNBC cells *in vitro* and tumor formation in xenograft mouse models. Moreover, inhibition of TNK2 with small molecule inhibitor (*R*)-9bMS significantly compromised TNBC proliferation.

Finally, we find that high levels of TNK2 expression in high-grade basal-like breast cancers correlates significantly with poorer patient outcome. Taken together, our study suggests that TNK2 is a novel potential therapeutic target for the treatment of TNBC.

## INTRODUCTION

Breast cancer is a heterogeneous disease, with major subtypes categorized by expression of estrogen receptor (ER), progesterone receptor (PR) and HER2 receptor. Triple negative breast cancer (TNBC) is a subgroup of breast cancer cases that lack the expression of all these three receptors. TNBC accounts for ~15% of invasive breast cancers and these cancers are often highly proliferative, poorly differentiated and associated with poor prognosis [1-4]. Unlike ER positive luminal breast cancers or breast cancers with HER2 amplification that can be treated with endocrine therapy [5] or HER2-targeted therapy [6], no targeted therapy is currently available for patients with TNBCs. A recent study found that several receptor tyrosine kinases including EGFR, MET and c-Kit were transcriptionally upregulated in different subsets of TNBCs [7, 8], suggesting that these upregulated receptor tyrosine kinases in TNBCs might be potential therapeutic targets for the treatment of TNBCs. However, clinical trials of targeting receptor tyrosine kinases in TNBCs with specific kinase inhibitors have, to date, produced mainly discouraging results. For example, treatment with monoclonal anti-EGFR antibody cetuximab, alone or in combination with cytotoxic chemotherapies, showed minimal improvement in progression-free and overall survival of TNBC patients [9, 10]. These data suggest that molecules other than receptor tyrosine kinases may be involved in driving TNBCs or that one needs to further stratify the patients based on expression of individual molecular markers.

In our previous efforts to uncover the role of tyrosine kinase signaling pathways that are etiologically activated in TNBCs, we used quantitative mass spectrometry-based phosphoproteomics to globally profile the phosphotyrosine proteomes of a panel of 26 TNBC cell lines, leading to identification and quantitation of over 2,500 phosphopeptides [11]. Through systematic phenotypic characterization using invasion and soft agar colony formation assays of each cell line, we found that these TNBC cell lines were highly heterogeneous in terms of their growth rates and invasiveness. By correlating our phosphoproteomic data with oncogenic phenotypes, we identified the non-receptor tyrosine kinase, TNK2, as a protein that was hyperphosphorylated in a majority of aggressive TNBC cell lines, suggesting that TNK2 is a regulator of TNBC growth and proliferation.

TNK2, also known as ACK1 (activated Cdc42-associated kinase), is a non-receptor tyrosine kinase which has been shown to be frequently amplified or mutated in multiple human cancers including breast, esophageal, lung, ovarian, pancreatic and prostate cancer [12, 13]. Overexpression of TNK2 in cancer cell lines can increase the invasive phenotype, both *in vitro* and *in vivo*, resulting in increased mortality of a mouse model of metastasis [13, 14]. Clinically, it was observed that amplification of the *TNK2* gene in primary tumors correlates with poor prognosis [13]. Further, expression of activated TNK2 was positively correlated with the severity of disease progression, and inversely correlated with the survival of breast cancer patients [15]. Being a cytoplasmic kinase, TNK2 couples extracellular signals from multiple receptor tyrosine kinases including EGFR, HER2 and PDGFR to intracellular effectors [15-18].

It has been suggested previously that phosphorylation of TNK2 might correlate with breast cancer progression [15, 19]; however, the functional significance of TNK2 expression and its role in breast cancer biology — particularly in TNBCs — has not been well elucidated. Based on emerging importance of TNK2 in various malignancies, we undertook detailed characterization of this relatively understudied non-receptor tyrosine kinase. In the current study, we show that TNK2 is overexpressed in a majority of TNBC cell lines and TNK2 expression level is significantly associated with the aggressiveness of TNBCs. By performing genetic ablation studies, we have demonstrated that TNK2 expression not only drives TNBC proliferation but also confers invasiveness, which is reflected in tumor formation in xenograft mouse models. Further, analysis of breast cancer patient survival data revealed that high TNK2 expression is significantly correlated with worse outcome of high-grade TNBC patients. In addition, the importance of TNK2 signaling was also reflected in cell proliferation studies; treatment with TNK2-specific small molecule inhibitor, (*R*)-**9b**MS, significantly suppressed growth of multiple TNBC cell lines. Taken together, these data uncover dependence of TNBCs on TNK2 signaling for both proliferation and invasion capabilities, and offer the promise of TNK2 inhibition as a therapeutic strategy for a subset of aggressive TNBCs.

## RESULTS

### Phenotype analyses correlate TNK2 levels with cellular aggressiveness

**Figure 1:**
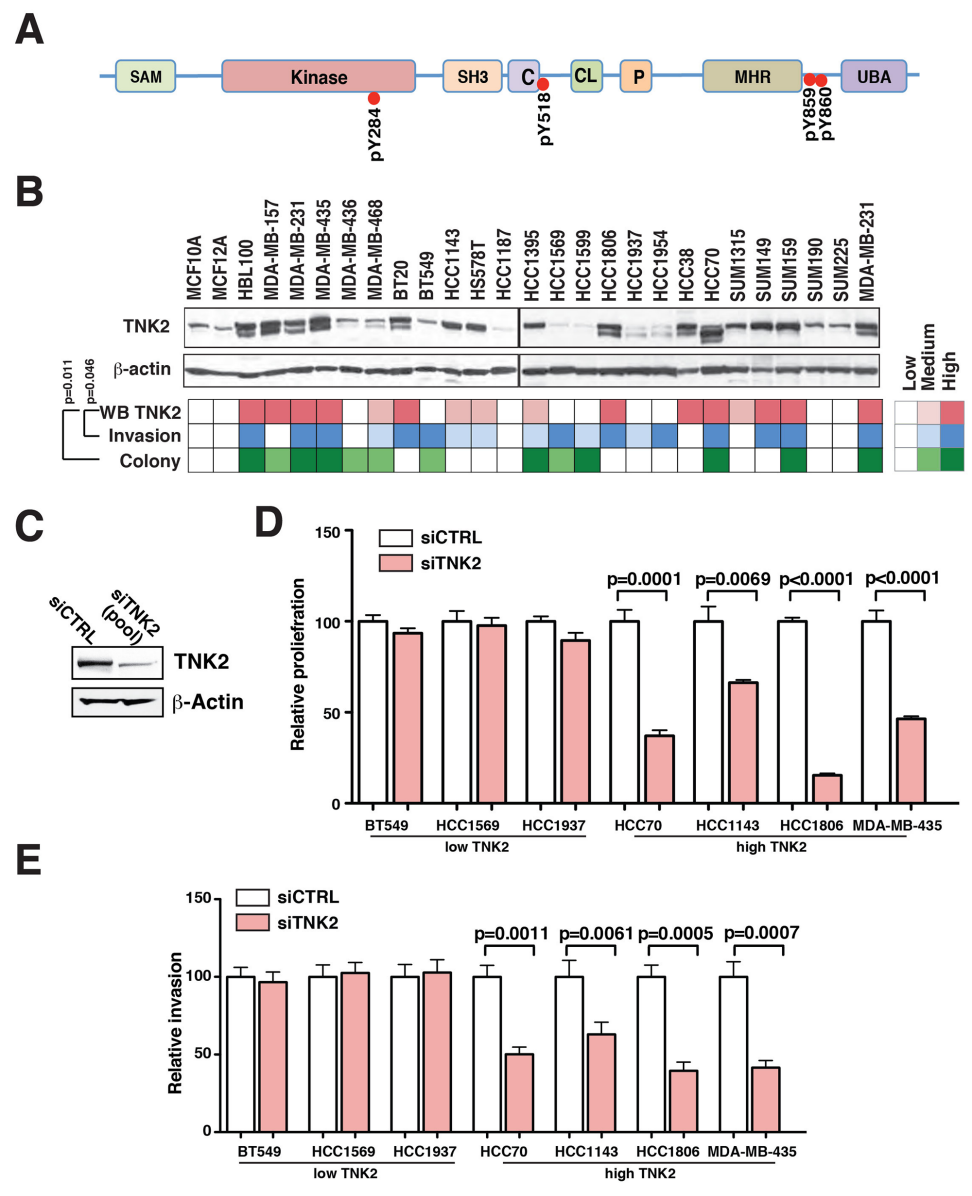
TNK2 is overexpressed in aggressive TNBC cell lines and required for oncogenic phenotype **A.** A schematic representation of TNK2 domain architecture. Different structural domains present in TNK2 are shown. C, Cdc42-binding domain; CL, clathrin-interacting domain; kinase, tyrosine kinase domain; P, PPXY motif or WW domain-interacting region and SAM, sterile α motif. Tyrosine phosphorylation sites identified in our global phosphoproteomic study are specified. **B.** TNK2 expression level correlates with the aggressive phenotype of TNBC cells. Top panel: Western blot analysis to detect expression of TNK2 in the panel of TNBC cell lines. Color-coded plots showing the expression level of TNK2 (top row), invasiveness (middle row) and colony formation ability (bottom row) across the panel of TNBC cells. Spearman's rank correlation was performed for statistical analysis. **C.** TNK2 siRNA knockdown efficiency was examined in HCC1395 cells. 50 nM siRNA were transfected in HCC1395 cells and an immunoblot was performed to evaluate the knockdown efficiency. β-Actin serves as the loading control. Proliferation **D.** and invasion assay **E.** after knockdown using siRNA against TNK2 (siTNK2) or control siRNA (siCTRL) in TNBC cells that have low expression of TNK2 (BT549, HCC1569, HCC1937) or high expression of TNK2 (HCC70, HCC1143, HCC1806, MDA-MB-435).

In our previous study characterizing the activated tyrosine signaling pathways of 26 TNBC cell lines [11], we identified four phosphotyrosine sites of TNK2 (pY284, pY518, pY859 and pY860) in multiple TNBC cells lines (Figure 1A) and discovered that TNK2 pY518 is hyperphosphorylated in a majority of aggressive cell lines [11]. In order to examine the expression level of TNK2 in TNBC, we performed immunoblotting for TNK2 in our panel of 26 TNBC cell lines (Figure 1B). A significant fraction (15 out of 26) of TNBC derived cell lines exhibited medium to high levels of TNK2 expression. When we examined how TNK2 expression might be related to anchorage-independent growth and cellular invasion (as determined by colony formation and matrigel invasion assays, described in our previous study [11]), we observed that TNK2 expression levels were significantly correlated with anchorage-independent growth and cellular invasiveness, suggesting that TNK2 plays an important role in regulating oncogenicity of TNBC cells (Figure 1B). Interestingly, HBL100, one of the immortalized non-tumorigenic mammary epithelial cell line, shows a relative aggressive phenotype and high expression of TNK2 in our study. It has been shown that HBL100 cells express the oncogenic SV40 large T antigen [20]. Similar aggressive phenotype of HBL100 has also been reported in other studies [21, 22].

To further test the role of TNK2 in regulating tumorigenicity, we performed siRNA knockdown of TNK2 in four TNBC cell lines with high expression levels of TNK2 (MDA-MB-435, HCC1806, HCC70 and HCC1143) and three cell lines with low levels of TNK2 expression (BT549, HCC1937 and HCC1569). The SMARTPool siRNA containing four different siRNA targeting different TNK2 mRNA regions was able to efficiently knockdown TNK2 expression (Figure 1C). We then performed invasion and proliferation assays on these seven manipulated cell lines and found that, in cells expressing high TNK2 levels, proliferation and invasion of cells expressing high TNK2 levels were significantly reduced by knockdown of TNK2. However, the proliferation and invasiveness of the cell lines expressing low levels of TNK2 were not affected with TNK2 knockdown (Figure 1D, 1E and Supplementary Figure S1). These results suggest that high-expression/activation of TNK2 plays an important role in regulating aggressive phenotype of TNBC cells.

### TNK2 knockdown reduces aggressiveness of TNBC *in vitro*

**Figure 2:**
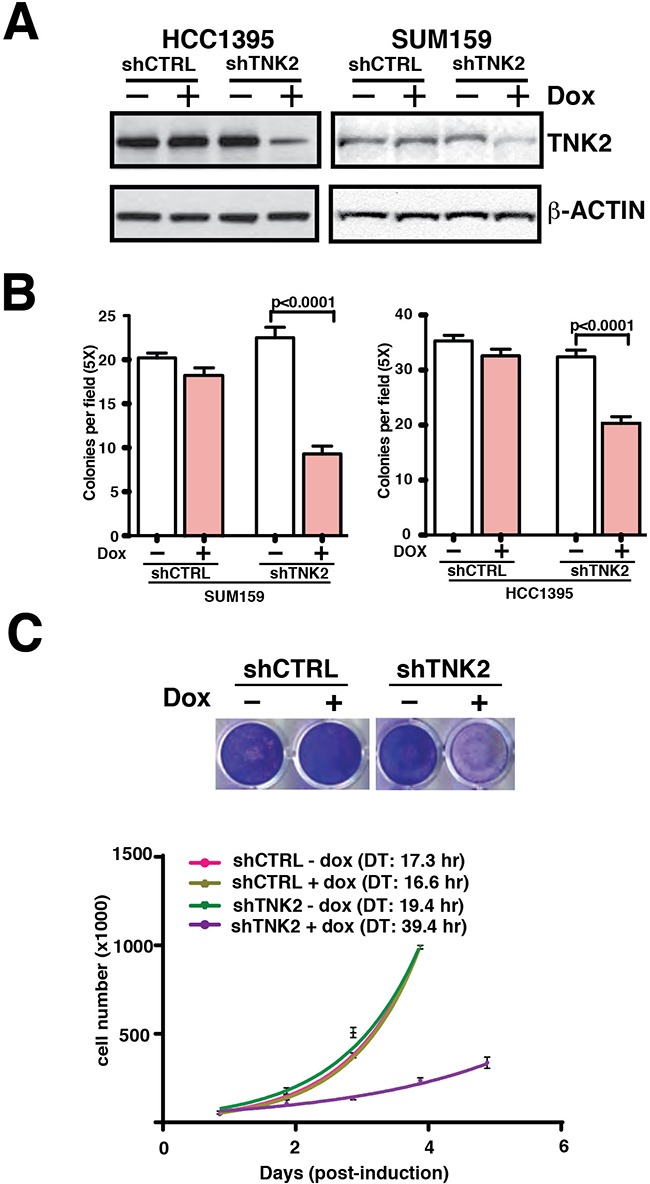
Inducible shRNA knockdown of TNK2 suppresses TNBC oncogenic phenotypes in vitro **A.** Western blot analysis to examine TNK2 expression in HCC1395 and SUM159 cells with inducible TNK2 shRNA or scrambled control shRNA. 100 ng/ml doxycycline was used for induction. **B.** Colony formation assays with shTNK2/shCTRL transfected SUM159 and HCC1395 cells with or without doxycycline induction. Mann-Whitney tests were performed for statistical analyses. **C.** Cell proliferation assays with shTNK2 and shCTRL HCC1395 transfected cells with or without doxycycline induction. The cells were stained with crystal violet to visualize the difference in the number of cells (top) or the doubling time (DT) calculated for each condition (bottom).

Dependence of TNBCs on TNK2 signaling opens up the possibility that genetic ablation of TNK2 in these cells could suppress their proliferation and invasion. To address the role of TNK2 in aggressive breast cancers, we established a lentiviral-based Tet-ON inducible shRNA knockdown system to suppress TNK2 expression in a regulated fashion. The shTNK2 and scrambled control shRNA (shCTRL) expressing lentiviral particles were then used to infect HCC1395 and SUM159 cells—two highly aggressive TNBC cell lines with high basal expression of TNK2. Regulated induction of TNK2 knockdown with doxycycline was determined to be efficacious (Figure 2A, top panel). Upon induction by doxycycline, HCC1395-shTNK2 and SUM159-shTNK2 cells showed significant reduction in colony formation in soft agar (Figure 2B). To calculate cell-doubling time for HCC1395 cells with or without induction of shTNK2, we performed quantitative cell proliferation assays. As shown in Figure 2C, almost no difference in cell proliferation was observed in HCC1395-shCTRL cells regardless of doxycycline induction. However, doxycycline induction of HCC1395-shTNK2 cells substantially prolonged HCC1395 doubling time by >2-fold.

**Figure 3:**
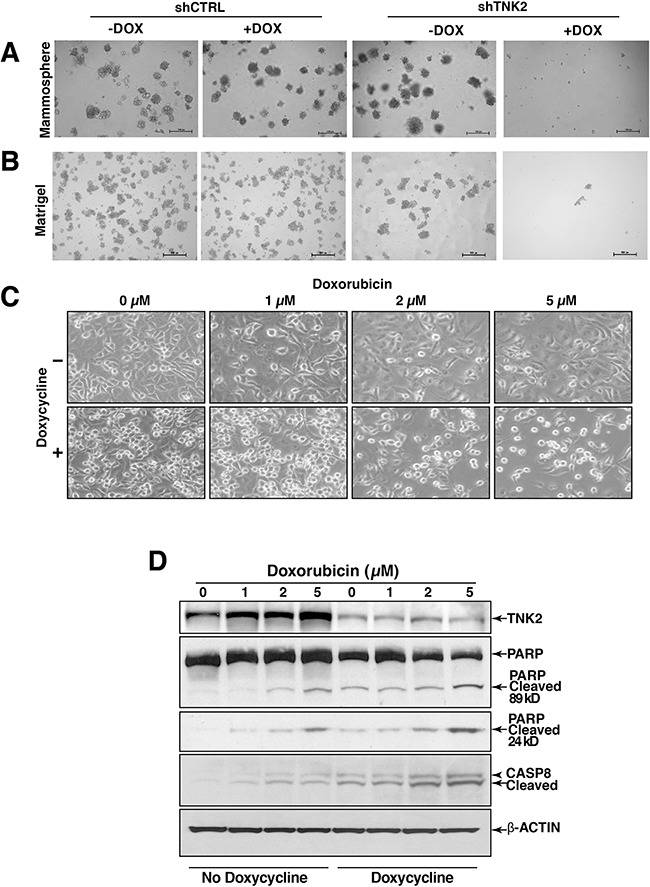
TNK2 is required for mammosphere formation Mammosphere assays **A.** and 3D matrigel assays **B.** of shTNK2/shCTRL HCC1395 cells with or without doxycycline induction. **C.** Synergistic effect of knocking down TNK2 with doxorubicin treatment. Doxycycline was used to induce shTNK2 expression. Induced or uninduced cells were treated with different doses of doxorubicin (as indicated). **D.** Western blot analysis to examine cleaved PARP and Caspase 8 in shCTRL and shTNK2 cells treated with doxorubicin and with or without doxycycline induction of shRNA. β-Actin serves as a loading control.

Subsequently, we studied the role of TNK2 on mammosphere formation, which is commonly used to indicate cellular stemness, an important tumorigenic hallmark in breast cancer. We found that upon induction of TNK2 shRNA, the cells completely lost their ability to form mammospheres (Figure 3A). This indicates that TNK2 expression is important for these cells to maintain their stem cell-like properties. To assess the potential effects of targeting TNK2 *in vivo*, we also performed 3-D Matrigel culture assays to better mimic *in vivo* physiological conditions. Similar to our mammosphere assay results, we observed that induction of TNK2 knockdown in HCC1395-shTNK2 cells completely eradicated colonies formed in the matrigel compared to HCC1395-shTNK2 with no induction and to HCC1395-shCTRL cells (Figure 3B), supporting our hypothesis that TNK2 expression/activation is important for TNBC tumorigenesis *in vivo*.

We then tested whether knocking down TNK2 provided any synergistic activity with doxorubicin, a common chemotherapy agent. We observed that cells with TNK2 knock down by shRNA became rounder and started to die at a much lower dose of doxorubicin compared to cells that were treated with the same concentration of doxorubicin but were not induced to express TNK2 shRNA, which showed a more normal morphology (Figure 3C). To determine if cells were undergoing apoptosis due to these treatments, we performed western blots to examine the apoptosis markers including the cleaved poly(ADP-ribose) polymerase 1 (PARP) and Caspase 8. As shown in Figure 3D, the levels of cleaved PARP and Caspase 8 were substantially increased in shTNK2 knockdown cells treated with doxorubicin compared to the cells without shTNK2 induction. Taken together, these results suggest a synergistic effect in reducing TNK2 expression with the treatment of chemotherapy agents such as doxorubicin.

### TNK2 knockdown reduces aggressiveness of TNBC *in vivo*

**Figure 4:**
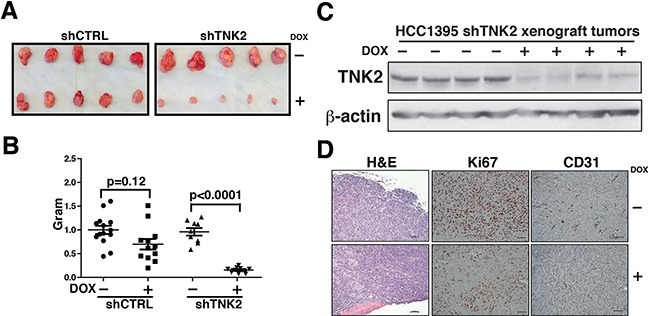
TNK2 knockdown suppresses tumor formation **A.** Tumors harvested from mice injected with HCC1395-shTNK2 cells or HCC1395-shCTRL cells with or without doxycycline induction. **B.** Weight of tumors resected from mice xenografted with HCC1395-shCTRL or HCC1395-shTNK2 cells with or without doxycycline induction. P-values from Mann-Whitney tests to measure the statistical significance of size differences across the indicated groups are shown. **C.** Expression of TNK2 or beta actin detected by western blotting using specific antibodies in HCC1395 xenografted tumors is shown for the various tumors. **D.** Hematoxylin and eosin staining or immunohistochemical labeling for Ki67 and CD31 in HCC1395- shTNK2 xenografted tumors with (+) or without (-) induction of shTNK.

To test the role of TNK2 in tumor growth *in vivo*, we established orthotopic xenograft using Tet-ON shTNK2 or shCTRL HCC1395 cells transplanted into mammary fat pads of NOD-SCID mice. shRNA (shTNK2 or shCTRL) expression was induced *in vivo* by doxycycline dissolved in drinking water. As shown in Figure 4A, tumor size in the doxycycline-induced shTNK2 group was significantly reduced as compared to the control group (Figure 4A, 4B). Western blot analysis for TNK2 protein expression confirmed the decrease in TNK2 expression in doxycycline-induced shTNK2 xenograft tumors (Figure 4C). Histopathological examination of the non-induced shTNK2 tumors demonstrated a lack of well-defined capsular margins, with cells pushing the borders for invasion compared to the shTNK2-induced tumors (Figure 4D). Immunohistochemical examination of tumor sections with Ki67, a marker of proliferation, and CD31, a marker of vascular endothelial cells, revealed that the shTNK2-induced tumors indeed showed significantly lower extent of proliferation and vascularization (Figure 4D). Overall, our data shows that TNK2 plays important roles in conferring oncogenic phenotype in aggressive TNBCs *in vivo*.

### TNK2 levels correlate with poor outcome in patients

**Figure 5:**
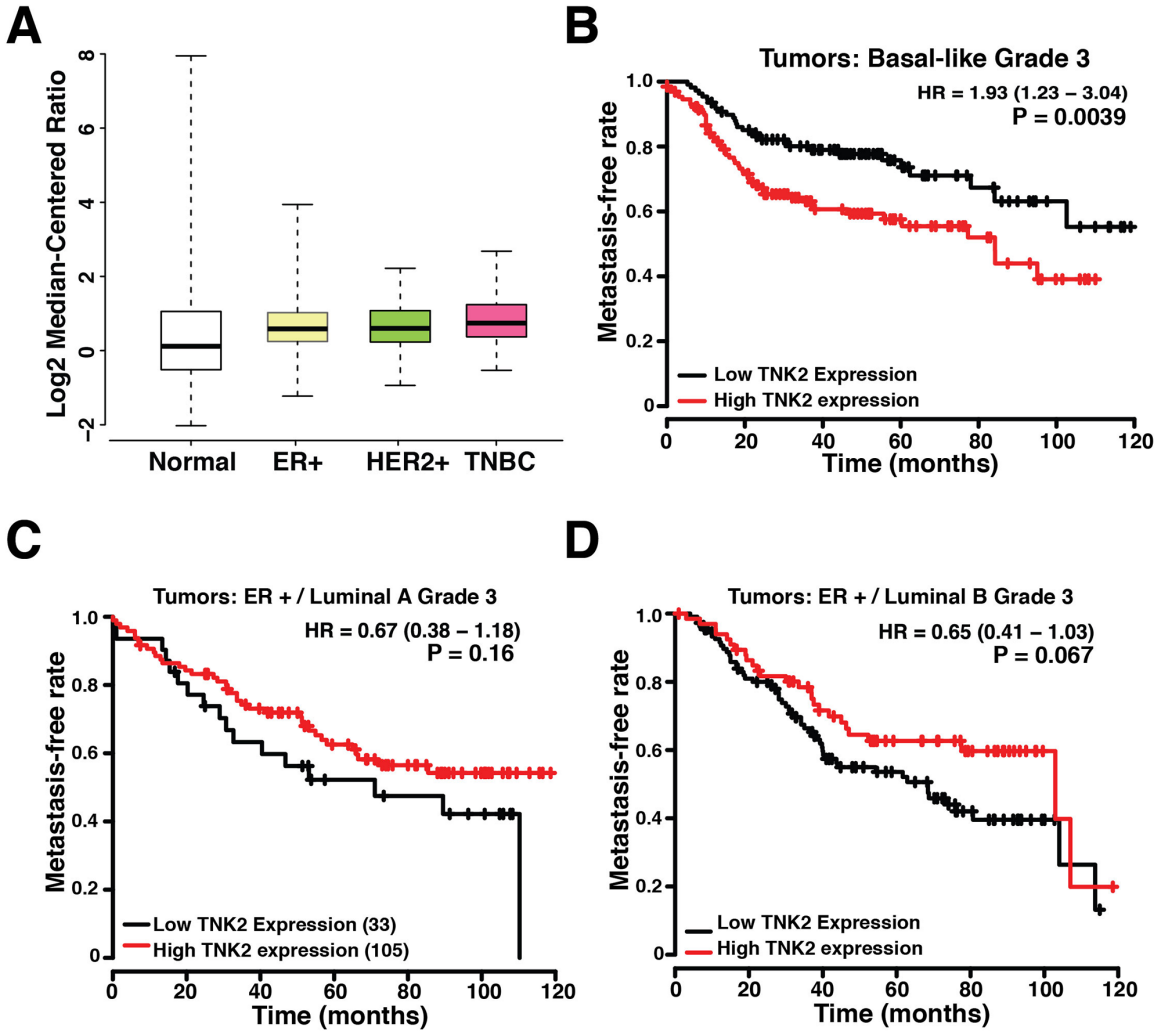
TNK2 levels correlate with poor outcome in patients **A.** TNK2 expression level in normal breast and different subtypes of breast cancer. Expression data were retrieved from Oncomine using TCGA dataset and re-plotted with R package. **B.** Kaplan-Meier plot of 237 high-grade basal-like breast cancer patients stratified with high or low *TNK2* gene expression. **C-D**. Kaplan-Meier plots of 138 high-grade luminal A (C) and 184 luminal B (D) breast cancer patients. The red line represents the survival curve of patients with high expression of TNK2 and the black line represents the surviving curve of patients with low expression of TNK2.

To address the clinical relevance of TNK2 expression, we first examined the TNK2 expression levels in TCGA dataset using Oncomine concept analysis. As shown in Figure 5A, TNK2 mRNA expression level is lowest in normal breast and highest in TNBC comparing to other ER+ luminal and HER+ breast cancers. We also performed survival analysis using a publicly available gene expression database of breast tumors from 4,142 patients [23]. With our analysis focusing on the 669 tumors that were classified as basal-like breast cancers, we found no significant correlation between TNK2 expression and patient survival. However, when a subpopulation of 237 high grade basal-like breast cancer tumors were selected for the analysis, high TNK2 expression levels were significantly associated with poorer patient outcomes (p<0.01) (Figure 5B). It is also worth noting that TNK2 expression did not show a statistically significant correlation with clinical outcomes for patients with high grade ER-positive tumors (Figure 5C, 5D). We believe this clinical result when added to the results from our *in vitro* and *in vivo* studies provides strong evidence to support identifying TNK2 as a novel target for personalized therapy for patients with basal-like/triple negative breast cancers that express high levels of this protein kinase.

### TNK2 kinase inhibition by small molecule inhibitor suppresses TNBC proliferation

**Figure 6:**
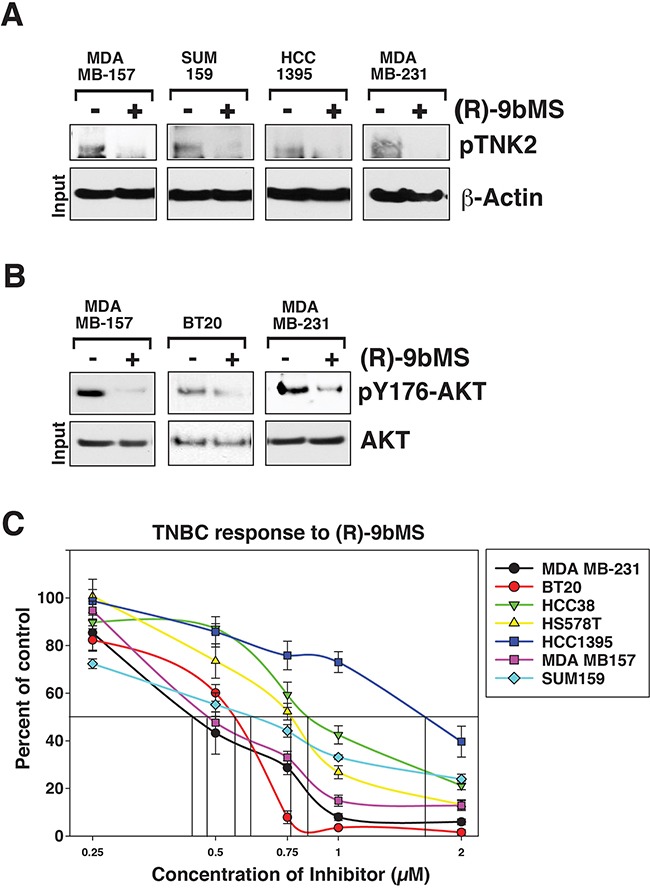
Small molecule inhibitor (R)-9bMS sensitizes TNK2 and its substrate AKT kinase phosphorylation compromising TNBC proliferation **A.** TNBCs were treated with TNK2 inhibitor (5μM, 24 hours); lysates were immunoprecipitated with TNK2 antibodies, followed by immunoblotting with pTyr-antibodies. **B.** TNBC were treated with TNK2 inhibitor (5μM, 24 hours); lysates were immunoprecipitated with pY176-AKT antibodies, followed by immunoblotting with pan AKT-antibodies **C.** TNK2 inhibitor (*R*)-**9b**MS reduces TNBC growth. Cells were treated with (*R*)-**9b**MS for 96hr and the number of viable cells were counted by trypan blue exclusion assay. Data represented as mean ± SEM (*n* = 2, three replicates).

Ability of TNK2 to directly phosphorylate AKT at Tyr176 site leading to its activation [15] indicates that TNK2-AKT signalling nexus may be involved in driving TNBC tumorigenicity. Recently, we have reported a new class of robust and soluble TNK2–specific small molecule inhibitor, (*R*)-**9b** MS [24, 25]. To address the role of TNK2 in AKT Tyr176-phosphorylation in TNBCs, cells were treated with (*R*)-**9b**MS. A significant decrease in TNK2 activation, as seen by loss of TNK2 Tyr-phosphorylation is observed upon (*R*)-**9b**MS treatment (Figure 6A). Inhibition of TNK2 activation is also reflected in significant decrease in AKT Tyr176-phosphorylation (Figure 6B). Further, several TNBC cell lines were found to be sensitive to the (*R*)-**9b**MS treatment with IC50 of 0.45 μM (MDA-MB-231), 0.48 μM (MDA MB 157), 0.6 μM (BT20), 0.67 μM (SUM159), 0.75 μM (HS578T) and 0.8 μM (HCC38) and 1.75 μM (HCC1395) (Figure 6C). Taken together, these data suggest that TNK2-AKT signaling may play an important role in TNBC growth and proliferation.

## DISCUSSION

In our previous study, we investigated the intrinsic heterogeneous signaling networks present within the TNBC cell population [11]. In this study, as an extension to our previous work, we focused on a non-receptor tyrosine kinase, TNK2, which is an important signaling molecule involved in integrating signals from a number of different receptor tyrosine kinases [12]. Although TNK2 has also been reported to be amplified and mutated in multiple cancers [12], in breast cancer, its activation is likely to be facilitated by receptor tyrosine kinases that correlate with disease progression as well as survival.

Our global phosphotyrosine study identified several tyrosine phosphorylation sites in TNK2 (Figure 1A) in a number of TNBC cell lines and phosphorylation level of pY518 is elevated in TNBC cells with aggressive phenotypes [11]. The current study has revealed that TNK2 is not only hyperphosphorylated, but also overexpressed in many highly aggressive human TNBC tumors and also in TNBC cell lines, and that the expression of TNK2 correlates with aggressive phenotypes of TNBC cell lines. We also demonstrated that targeting TNK2 with specific siRNA/shRNA can substantially attenuate oncogenic phenotypes of TNBC cells *in vitro* and, more importantly, dramatically reduce tumor formation in preclinical xenograft mouse models, suggesting that these aggressive characteristics are driven by TNK2. Finally, our study shows promise for combining anti-TNK2 therapy with doxorubicin treatment for aggressive TNBCs, providing a pathway for future clinical applications of our findings.

Recent studies have shown that several ubiquitin ligases including NEDD4-1 [26], NEDD4-2 [27], SIAH1 and SIAH2 [28] can ubiquitinate TNK2 and induce TNK2 degradation. Of note, in ER positive breast cancer cells, estrogen can activate ER and induce SIAH2 expression that subsequently ubiquitinates TNK2 and reduces TNK2 expression. Absence of ER in TNBC could be a potential reason why TNK2 is more expressed in TNBC (Figure 5A). A major downstream effector of TNK2 in hormonally regulated cancers has been AR [29], which also points to the potential of ER as another TNK2-interacting entity. However, TNBCs present a conundrum as although these cells lack expression of ER, they are exquisitely sensitive to loss of TNK2, suggesting that some other downstream effectors may be operational in TNBCs. TNK2 has been shown to interact with and directly phosphorylate AKT at an evolutionarily conserved tyrosine 176 residue, promoting AKT activation. Detection of AKT Tyr176-phosphorylation in TNBCs (Figure 6A), its sensitivity to TNK2 inhibitor (*R*)-**9b**MS which in turn compromising proliferation of TNBCs (Figure 6B) indicate that TNK2-AKT signaling may drive TNBC tumor growth. Future studies may reveal whether sensitization of TNBCs by TNK2 inhibitor also involves other TNK2 effectors.

About 10-20% of breast cancers are found to be triple-negative. Due to limited treatment options that are currently available, there is intense interest in finding new therapies that can treat this subtype of breast cancer. This study not only provides evidence for ‘addiction’ of TNBCs to TNK2 signaling, but also provides a rationale for exploring TNK2-specific small molecule inhibitor (*R*)-**9b**MS as a realistic treatment option for TNBC patients.

## MATERIALS AND METHODS

### Cell culture

Cell lines were obtained from ATCC and cultured in the appropriate media as previously described [11, 30, 31].

### Western blot and siRNA knockdown

Each cell line was harvested and lysed in modified RIPA, and immunoblotting was performed as previously described [32]. The primary antibodies used in this study are anti-TNK2 (Santa Cruz Biotechnology), anti-PARP cleaved 89 Kd protein (Cell Signaling), anti-PARP cleaved 24 kD protein (Abcam), anti-cleaved Caspase 8 (Cell Signaling) anti-AKT (Cell Signaling), and anti-pY176-AKT antibody that was developed in our previous study [15]. 50 nM siRNA targeting TNK2 (L-003102, SMARTpool, Dharmacon) was used for transfections with RNAiMax (Invitrogen). Cells were harvested 48 hours post-transfection for assessing knockdown efficiency or other follow-up experiments.

### Matrigel invasion assays

Cells were washed once with PBS, detached using trypsin (Life Technologies) and 5x10^4^ cells were seeded into Biocoat matrigel invasion chambers (BD Biosciences). Growth media supplemented with serum for each cell line was added in the lower wells as the chemoattractant. After 24 hours, the filter membranes were stained with DAPI (Invitrogen). The number of cells that penetrated through the matrigel and membrane was counted for ten randomly selected viewing fields at 20x magnification. ImageJ was used to count cell nuclei. Student's t-test was performed for statistical analysis.

### Soft agar colony formation assays

Briefly, 0.5 ml of 0.8% bottom layer agar was prepared in six-well plates. Cells with different treatments were separately trypsinized, centrifuged, resuspended in 0.4% agar medium (equal volumes of 0.8% agar and culture medium), and plated onto the top agar at 500 cells per well. The cells were grown for 14 days at 37°C. Colonies were then stained with crystal violet and counted under the microscope. Mann-Whitney tests were performed to for statistical analyses.

### MTT cell proliferation assay

MTT (3-(4, 5-dimethylthiazol-2-yl)-2, 5-diphenyl-tetrazolium bromide) assays were performed to measure cell proliferation. Briefly, cells that were transfected with different siRNAs in 96-well plate were left to grow for 5-7 days before the MTT assay. 1 mg/ml MTT in growth media was added into each well and the plate was incubated for two hours in 37°C. Media was then removed and 100 μl of DMSO and ethanol (1:1 by volume) was added into each well. The plate was then read for absorption at 530 nm on a microplate reader. Student's t-test was performed for statistical analysis.

To determine effect of TNK2 inhibitor, cells were treated with different concentration of (*R*)-**9b**MS for 96 hours and live cells (determined by trypan blue exclusion) were counted.

### Generation of inducible shTNK2 cells

A shRNA construct targeting a different TNK2 sequence (GGCAGUCAGAUCCUGCAUAAG) from that targeted by siRNA was designed and cloned into the pLKO-Tet-On-puro plasmid vector (Addgene) and the sequence was confirmed by Sanger sequencing. The plasmid was then packaged into viral particles by transfection into HEK293T cells along with packaging plasmids, psPAX2 and pMD2.G. After overnight transfection, the conditioned medium was collected and used to infect SUM159 and HCC1395 cells. Stably infected cells were selected by puromycin.

### *In vivo* tumor xenograft assays

NOD-SCID mouse xenografts were performed as previously reported [33]. Briefly, 20 four to six week old female NOD-SCID mice were purchased from NCI. HCC1395 cells stably infected with Tet-ON inducible TNK2 shRNA or control shRNA were resuspended in matrigel:PBS (1:1 volume). 5×10^5^ cells were injected onto the mammary fat pads of NOD-SCID mice at two sites per mouse. Mice were then either administered doxycycline in drinking water (2 mg/ml) to induce the expression of shRNA or normal water as control. Five mice were used for each treatment group. After four weeks, the mice were sacrificed and tumors were processed. Student's t test was performed for the statistical analysis to compare the tumor weight. All procedures were approved by Johns Hopkins University institutional Animal Care and Use Committee and were performed in accordance with the Animal Welfare Act regulations.

### Immunohistochemical labeling of xenograft tumors

Immunohistochemical staining was performed on the formalin-fixed, paraffin-embedded xenograft tumors. Briefly, paraffin embedded slides were rehydrated and then treated with hydrogen peroxide to block endogenous peroxidase activity, and then washed with PBST buffer. Slides were then blocked with goat serum before diluted rabbit polyclonal antibodies to Ki67 and CD31 were added for protein binding at 4°C and incubated overnight. The slides were washed with PBST, incubated with biotinylated secondary antibody (Dako), and treated with DAB substrate kit (Dako) according to the manufacturer's instructions. The slides were then counter-stained using Mayer's Hematoxylin (Dako) before visualizing under the microscope.

## SUPPLEMENTARY MATERIALS FIGURES


