# Supplementary material for: The non-receptor tyrosine kinase TNK2/ACK1 is a novel therapeutic target in triple negative breast cancer
Source: Oncotarget. 2016 Nov 25;8(2):2971–83. doi: 10.18632/oncotarget.13579 (PMC5356856; doi:10.18632/oncotarget.13579)
Supplement: Supplementary file 1 [file oncotarget-08-2971-s001.pdf]

The non-receptor tyrosine kinase TNK2/ACK1 is a novel therapeutic target in triple negative breast cancer

SUPPLEMENTARY FIGURE

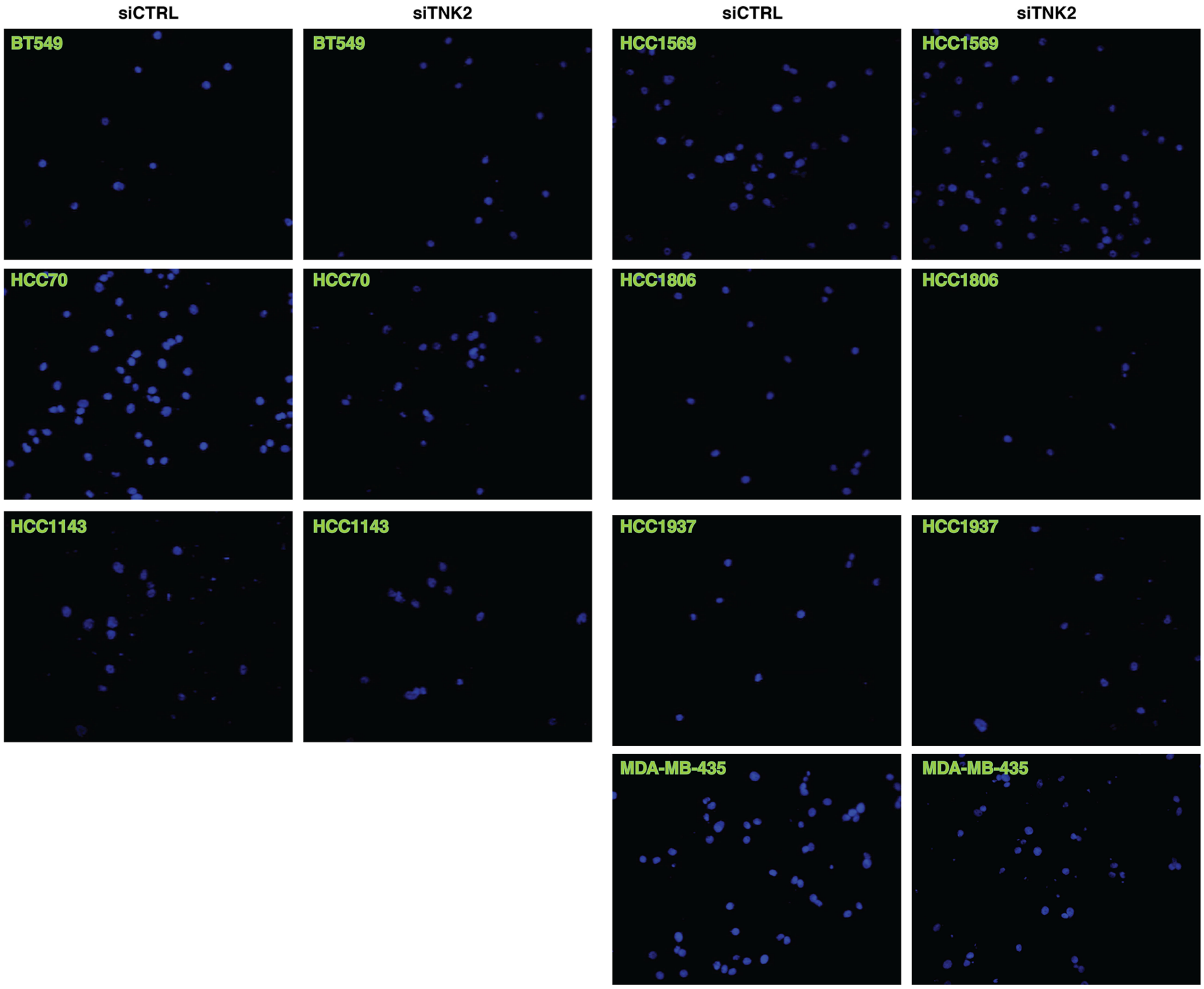

Supplementary Figure 1: Representative images of matrigel invasion assays for TNBC cells with siTNK2 knockdown. siRNA transfected TNBC cells (as indicated) were used for matrigel invasion assay. Cells invaded through the matigel and base membrane were stained with DAPI and images were taken with 20x objective lens using Nikon E400 microscope.
